# Effect of physical exercise on muscle strength in adults following bariatric surgery: A systematic review and meta-analysis of different muscle strength assessment tests

**DOI:** 10.1371/journal.pone.0269699

**Published:** 2022-06-10

**Authors:** Flávio Teixeira Vieira, Gabriela Sousa de Oliveira, Vivian Siqueira Santos Gonçalves, Silvia G. R. Neri, Kênia Mara Baiocchi de Carvalho, Eliane Said Dutra

**Affiliations:** 1 Graduate Program in Human Nutrition of the University of Brasilia, Brasilia, Brazil; 2 Graduate Program in Public Health of the University of Brasilia, Brasilia, Brazil; 3 Graduate Program in Physical Education of the University of Brasilia, Brasilia, Brazil; Federal University of Paraiba, BRAZIL

## Abstract

Individuals following bariatric surgery are considered at high risk for the development of sarcopenic obesity (excess fat mass, low muscle mass and low physical function), and exercise may play an important role in its prevention and treatment. We systematically reviewed 5 scientific databases (Embase, Medline, Scopus, SPORTDiscus, and Web of Science) and 2 grey literature databases (ProQuest and Google Scholar) for clinical trials that evaluated the effect of exercise on muscle strength in adults following bariatric surgery and conducted a separate meta-analysis for studies that used different muscle strength tests. Random-effect models, restricted maximum likelihood method and Hedges’ g were used. The review protocol was registered at the International Prospective Register of Systematic Reviews (PROSPERO) database (CRD42020152142). Fifteen studies were included (638 patients), none had a low risk of bias, and all were included in at least 1 of the 5 meta-analyses (repetition maximum [lower and upper limbs], sit-to-stand, dynamometer, and handgrip tests). Exercise interventions improved both upper (effect size, 0.71; 95% CI, 0.41–1.01; I^2^ = 0%) and lower (effect size, 1.37; 95% CI, 0.84–1.91; I^2^ = 46.14) limb muscle strength, as measured by repetition maximum tests. Results were similar for the sit-to-stand (effect size, 0.60; 95% CI, 0.20–1.01; I^2^ = 68.89%) and dynamometer (effect size, 0.46; 95% CI, 0.06–0.87; I^2^ = 31.03%), but not for the handgrip test (effect size, 0.11; 95% CI, -0.42–0.63; I^2^ = 73.27%). However, the certainty level of the meta-analyses was very low. Exercise with a resistance training component performed post bariatric surgery may improve muscle strength, which is related to sarcopenic obesity, functional capacity, and mortality risk, therefore should be included in the follow-up.

## Introduction

Bariatric surgery (BS) can lead to severe energy and protein restriction or malabsorption, particularly in the first year postoperatively, culminating in fat-free mass (FFM) loss [1, 2]. FFM is also associated with resting metabolic rate [3], longevity [4], and strength [5], which can be compromised during abrupt weight loss [6]. Individuals following BS are considered at high risk for the development of sarcopenic obesity (excess fat mass, low muscle mass and poor physical function) [7].

Regular physical activity is an important adjunct therapy following BS [8]. However, most individuals do not achieve minimum physical activity recommendations [9]. Previous meta-analyses have suggested that patients who perform exercise after BS demonstrate greater weight/fat loss and better aerobic capacity compared with sedentary patients [10, 11]. Furthermore, including resistance exercises in addition to aerobic exercises improved the results [10].

Aerobic exercise training has historically been associated with improved metabolic regulation, cardiovascular function, and aerobic capacity; however, it may also be associated with muscle hypertrophy [12]. Resistance training promotes muscle strengthening and induces muscle hypertrophy in the general population [13]. Although muscle mass and strength are positively correlated, comorbidities such as obesity may affect this association, due to muscle deconditioning, inflammation, and fat infiltration into muscle [14]. Exercise performed post BS struggles to generate changes in lean mass and may only exhibits increase in muscle strength (MS) [11, 15]. MS has a better prognostic value than FFM in predicting worsening disability [16]. Furthermore, MS has an independent inverse association with mortality risk [17].

Previous systematic reviews have addressed some of the effects of exercise on MS in the postoperative period following BS; however, most did not include a meta-analysis [10, 18, 19]. Bellicha et al. [11] were the first to publish a relevant meta-analysis; however, they combined the results of studies that evaluated MS with different tests and muscle groups. In many musculoskeletal conditions, optimal muscle function is important regarding quality of life and rehabilitation, and the maximal MS an individual can produce in different tasks should be known to design a proper rehabilitation program [20]. Each measurement test evaluates different MS features, therefore combining them as a single variable could decrease the inference power and limit appropriate conclusions.

Evaluating differences in MS according to specific muscle groups and strength tests may provide a deeper understanding of the association between physical exercise and MS. This may facilitate the development of optimal exercise interventions and MS assessment protocols for postoperative care after BS. Therefore, we systematically reviewed the effect of exercise on MS in individuals following BS and conducted a separate meta-analysis for studies that used different MS tests.

## Materials and methods

### Protocol, registration, eligibility criteria

An extensive systematic review of the literature was performed and meta-analyses were conducted to summarize the scientific evidence. This systematic review was conducted following the Preferred Reporting Items for Systematic Reviews and Meta-Analyses (PRISMA) checklist [21]. The review protocol was registered at the International Prospective Register of Systematic Reviews (PROSPERO) database (CRD42020152142). Both files are available as supporting information.

Clinical trials were included if they 1) evaluated adults who underwent BS (mostly Roux-en-Y gastric bypass [RYGB] and sleeve gastrectomy [SG]) at any postoperative time point; 2) contained information about the type, frequency, and duration of exercise intervention; 3) evaluated MS (using any method); and 4) included a control group. Studies that exclusively evaluated specific populations with chronic diseases and exercise interventions administered in conjunction with an ergogenic resource were excluded. To reduce publication and retrieval bias, the search was not restricted by language, publication date, or publication status. This article does not contain any studies with human participants or animals performed by any of the authors.

### Procedures

The search strategy was evaluated by an expert researcher using the Peer Review of Electronic Search Strategies (PRESS) checklist [22]. The PICO strategy was used for the research question construction and evidence search. Details of the search strategies adapted for the different databases are shown in S1 Table.

Five scientific databases (Embase, Medline, Scopus, SPORTDiscus, and Web of Science) and 2 grey literature databases (ProQuest and Google Scholar) were systematically searched. Google Scholar was partially searched; only the first 200 relevant articles were screened. All databases were searched up to October 27, 2021. The Rayyan^®^ software program was used to remove duplicate references before screening [23].

Study selection was conducted in 2 phases. In the first phase, 2 reviewers independently screened the titles and abstracts of the retrieved references. Studies that did not meet the eligibility criteria were excluded. In the second phase, the full texts of the articles identified in the first phase were independently assessed by the same reviewers. Disagreements regarding study eligibility were discussed between the 2 reviewers to reach a consensus; a third reviewer made a final decision when necessary. The reference lists of the included studies were also manually searched for relevant articles.

Data were independently extracted by 2 reviewers and cross-checked. Disagreements were resolved through discussion and, when necessary, a consensus was reached with the assistance of a third reviewer. The following variables were extracted from the included studies: country, study design, study aim, patient characteristics, BS type, postoperative time, intervention and control group protocols, strength measures, and outcomes/main results.

Authors were contacted by e-mail in cases where clarification was required or data of interest were missing. If no response was received within 2 weeks, a second e-mail was sent. The reviewers made a final decision if there was no response after another 15 days.

Risk of bias assessments were conducted independently by the 2 reviewers using the Joanna Briggs Institute critical appraisal tools for randomized controlled trials [24]. Any discrepancies were resolved by consensus; if necessary, a third reviewer served as the arbitrator. The instrument consists of 13 questions that evaluate the possibility of bias in the design, conduct, and analysis of each study. The possible answers are yes, no, unclear, and not applicable. An answer of “no” for any item meant that the study was not considered to have an overall low risk of bias. The risk of bias assessment was not used as a criterion for study eligibility.

### Summary measures and data analysis

Outcome measurements (mean and standard deviation) for MS were extracted at baseline and follow-up for both the exercise and control groups. Meta-analyses were conducted using random-effects models and the restricted maximum likelihood method [25]. Differences in parameters between the control and intervention groups were estimated using Hedges’ g and its 95% confidence interval (CI) [26].

Heterogeneity of treatment effects between studies was evaluated using the Chi-square method (p<0.10) and the I^2^ statistic. Following the recommendations of the Cochrane Collaboration, heterogeneity was not considered important if I^2^ was <40% [25]. To investigate parameters influencing heterogeneity, we performed subgroup analyses to evaluate the effects of assessing different muscle groups and the type of MS assessment. A sensitivity analysis was also performed to account for the type of intervention. Because of the small number of studies included in each meta-analysis, it was not possible to assess publication bias using meta-regression [25]. All statistical analyses were performed with Stata (version 16.1, Stata Corporation, College Station, TX) using the “meta” command.

Two reviewers independently evaluated the certainty of evidence from each meta-analysis with the Grading of Recommendations, Assessment, Development and Evaluations (GRADE) approach [27]. Disagreements were discussed between the 2 reviewers until they reached a consensus. In the GRADE approach, the certainty of evidence is rated as high, moderate, low, or very low by evaluating 5 domains (risk of bias, inconsistency, indirectness, imprecision, and publication bias). The GRADEpro GDT 2020^®^ software program was used to prepare the summary of findings table, which included the downgrade justification for each level of certainty.

## Results

### Study selection and characteristics

**Fig 1:**
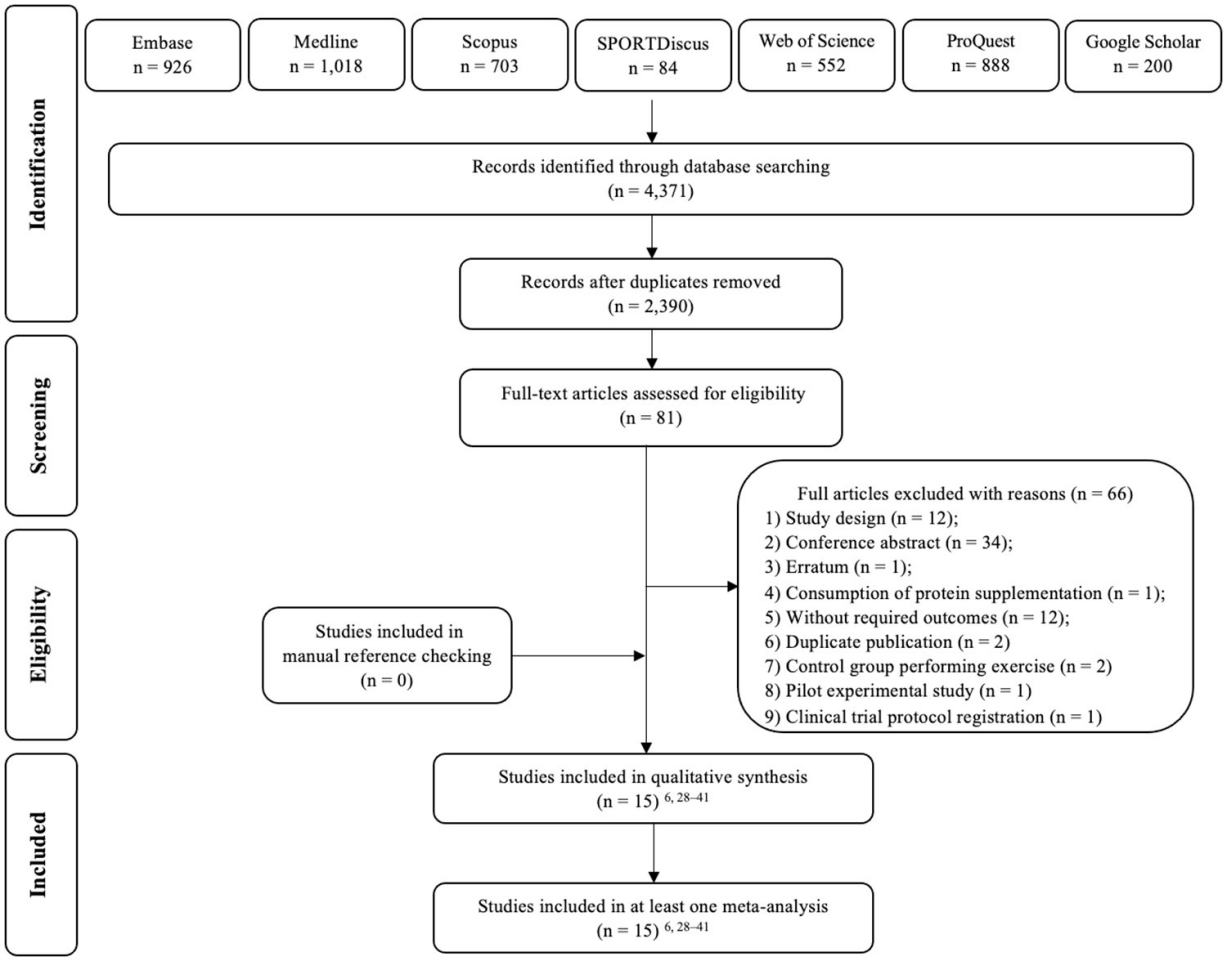
Flowchart of the selection process.

The literature search retrieved a total of 4371 studies. After the screening of titles and abstracts (phase 1), 81 potentially relevant studies were found. Phase 2 screening excluded 66 articles (Fig 1). Thus, 15 studies were included in this review, and all were included in at least 1 of the 5 meta-analyses [6, 28–41].

The included studies were published from 2011 to 2021 and were conducted in 9 different countries. The studies were randomized [6, 31–37, 39, 41] or nonrandomized [28–30, 38, 40] controlled trials (Table 1).

**Table 1:** Characteristics of included studies.

Study	Country	Study design	Aim of the study	Sample	Type of bariatric surgery	Postoperative time
Stegen et al., 2011	Belgium	NRCT	To investigate the effect of RYGB on physical fitness and to determine if an exercise program in the first 4 months is beneficial.	15 ♀♂ Age: 40.5 ± 8.1 BMI > 35	RYGB	1 month
Huck, 2015	USA	NRCT	To evaluate the feasibility of a 12-week supervised, resistance training program and its short-term effects on physical fitness and functional strength for this population	15 ♀♂ Age: 18–65 BMI: 37.7 ± 6.3	RYGB/GB	≤ 12 months
Campanha-Versiani et al., 2017	Brazil	NRCT	To evaluate bone mineral density and bone markers in a group submitted to a regular and supervised exercise program compared to a control group that did not perform exercises and evaluate muscular strength and body composition after 1 year of a combined program of weight-bearing and aerobic exercise, in obese patients who have undergone RYGB.	37 ♀♂ Age: 20–60 BMI > 40	RYGB	2 months
Coleman et al., 2017	USA	RCT	To conduct a pilot randomized trial testing an exercise program specifically adapted for post-bariatric patients.	44♀♂ Age: 49.8 ± 11.4 BMI: 30–35	RYGB/Sleeve/GB	6–24 months
Hassannejad et al., 2017	Iran	RCT	To evaluate the impact of aerobic and strength exercise after the bariatric surgery on weight loss and body composition outcomes and to investigate the improvement in functional capacity.	60 ♀♂ Age: 20–50 BMI > 35	RYGB/Sleeve	1 month
Herring et al., 2017	UK	RCT	To examine the effects of a supervised 12-week exercise intervention on physical function and body composition maintenance in patients who were between 12 and 24 months after bariatric surgery.	24 ♀♂ Age: >18 BMI > 30	RYGB/Sleeve/GB	12–24 months
Daniels et al., 2018	USA	RCT	To examine the effect of a 12-week resistance training programme on lean mass, muscle cross-sectional area, muscular strength and muscle quality in women who underwent RYGB surgery.	16 ♀ Age: 44.9±10.2 BMI NA	RYGB	2 months
Mundbjerg et al., 2018	Denmark	RCT	To investigate the effects of supervised physical training following RYGB on aerobic capacity, muscle strength and physical function 12–24 months post-surgery and furthermore to elucidate the effects of RYGB from pre-surgery to 6 months post-surgery on the same markers for physical capacity.	52 ♀ ♂ Age: 42.3±9.1 BMI 33.7±5.8	RYGB	6 months
Kelley, 2019	USA	RCT	To investigate the effects of eccentric exercise on lower body skeletal muscle mass during rapid body mass loss induced by bariatric surgery	13 ♀ Age: 37.9±8.1 BMI 39.3±4.3	RYGB/Sleeve	1–2 months
Noack-Segovia et al., 2019	Chile	RCT	To evaluate a physical exercise program of moderate intensity in patients operated of bariatric surgery and its influence on muscle strength.	43 ♀ ♂ Age: 33.0±6.9 BMI 35.5±3.3	NA	1 month
Gallé et al., 2020	Italy	NRCT	To evaluate the effects of an integrated post-operative exercise-based educational and motivational program implemented immediately after surgery on lifestyles, quality of life, anthropometry, cardiorespiratory fitness, muscular strength and flexibility respect to the only surgical intervention in a sample of Italian sedentary bariatric patients.	70 ♀ ♂ Age: 18–65 BMI 33.8±5.1	Sleeve/GB	≤ 6 months
de Oliveira Junior et al., 2021	Brazil	RCT	To investigate the impact of a home-based exercise training program in patients who had surgery and were provisionally deprived from in-hospital health care.	70 ♀♂ Age: 47.5±11.6 BMI: 36.0±6.8	RYGB/Sleeve	3–12 months
Diniz-Souza et al., 2021	Portugal	RCT	To investigate whether a supervised multicomponent exercise program could induce benefits on bone mass after bariatric surgery.	61 ♀ ♂ Age: 18–65 BMI ≥ 35	RYGB/Sleeve	1 month
Gil et al., 2021	Brazil	RCT	To comprehensively examine the effects of exercise training on body composition (fat-free mass as primary outcome), muscle function and related cellular and molecular mechanisms (secondary outcomes) in women undergoing bariatric surgery.	55 ♀ Age: 18–60 BMI ≥ 35	RYGB	3 months
Lamarca et al., 2021	Brazil	NRCT	To investigate the effects of resistance training with and without whey protein supplementation on body composition and Resting Energy Expenditure in the late postoperative period of RYGB.	63 ♀ ♂ Age: 40.3±8.3 BMI 29.7±5.3	RYGB	24–84 months

Age, years; BMI, Body mass index (Kg/m^2^); GB, Gastric banding; NA, Not available; Max, Maximum; NRCT, Non-randomized controlled trial; RCT, Randomized controlled trial; RYGB, Roux-en-Y gastric bypass; Sleeve, Sleeve gastrectomy; UK, United Kingdom; USA, United States of America. ♀ for female and ♂ for male.

The total number of patients across all studies was 638; individual study sample sizes ranged from 13 [36] to 70 [38] patients. Patient age ranged from 18–65 years, and the majority of patients had a body mass index >30 kg/m^2^. Three studies only evaluated women [6, 34, 36].

The distribution of surgery types was as follows: RYGB (n = 6) [6, 28, 30, 34, 35, 40]; RYGB and SG (n = 4) [32, 36, 39, 41]; RYGB and gastric banding (GB, n = 1) [29]; SG and GB (n = 1) [38]; and RYGB, SG, and GB (n = 2) [31, 33]. Noack-Segovia et al. [37] did not specify the type of BS. In most studies, the physical exercise intervention was initiated in the early postoperative period, either between 1 and 3 months [6, 28, 30, 32, 34, 36, 37, 39] or ≤6 months after surgery [38]. Two studies administered the exercise intervention up to 12 months after surgery [29, 41], whereas in 3 studies this was done between 6 and 24 months postoperatively [31, 33, 35]. Lamarca et al. [40] were the only study that included patients who underwent BS more than 2 years prior.

### Risk of bias

**Fig 2:**
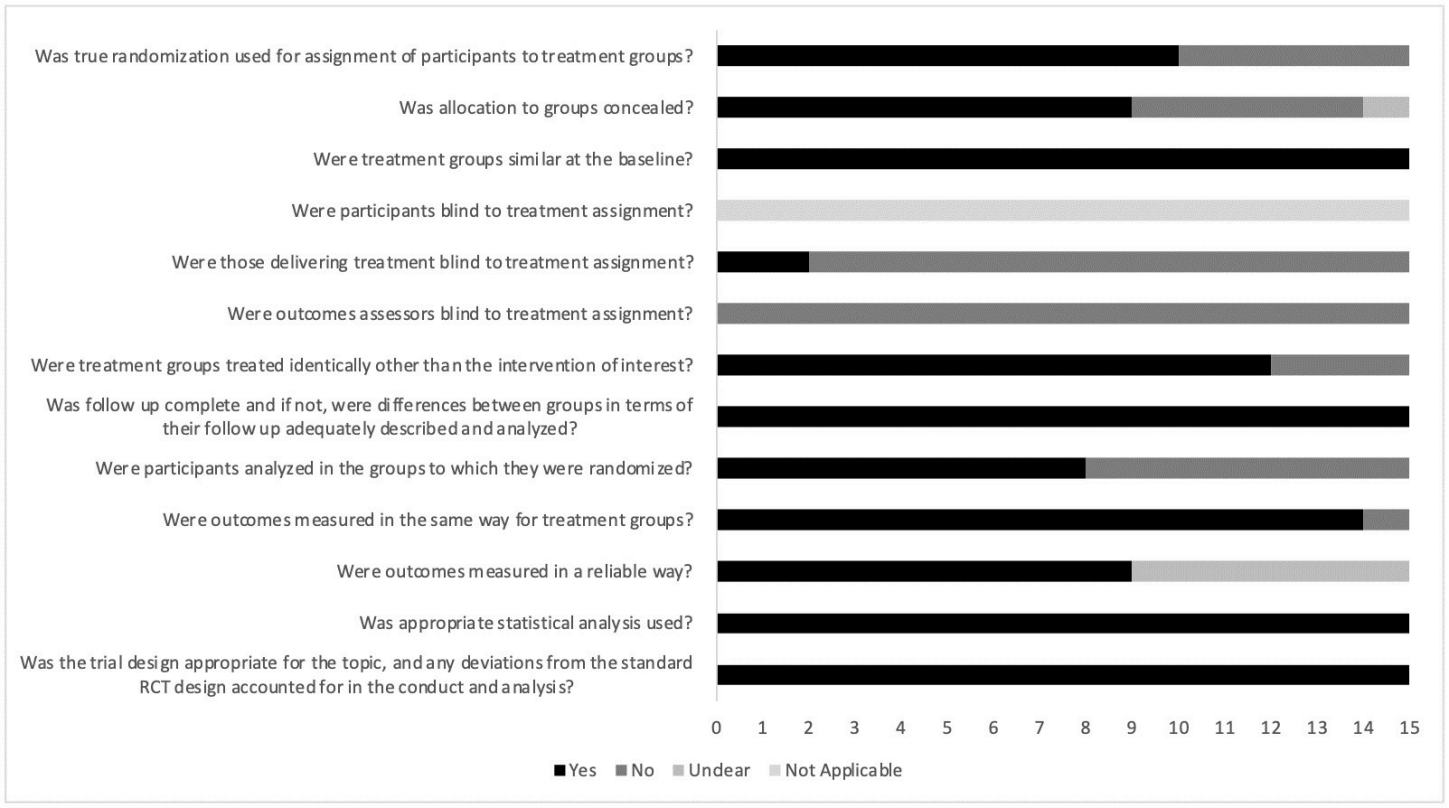
Risk of bias in the included studies (The Joanna Briggs Institute checklist for randomized controlled trials).

None of the studies had a low risk of bias (Fig 2, and S2 Table). Most studies did not specify the blinding of outcome assessors to treatment allocation [6, 28–41] or the blinding of exercise specialists who delivered the treatment [6, 28, 30–37, 39–41]. In contrast, all studies satisfied the following criteria: 1) similarity between groups at baseline; 2) complete follow-up (or adequate analysis of differences between groups in terms of their follow-up); 3) appropriate statistical analysis; and 4) appropriate trial design. Only Huck [29] did not assess outcomes in the same way for the treatment groups. As exercise is an intervention type that cannot be blinded for participants, all studies received a “not applicable” rating for this item.

### Intervention characteristics

Most studies combined aerobic and resistance training [6, 28, 30, 31, 33, 35, 37–39, 41], whereas 3 only utilized resistance training [29, 34, 40]. Two studies compared the effects of different interventions, in addition to a control group. Hassannejad et al. [32] compared the combination of aerobic and resistance training with aerobic training alone. In the study by Kelley [36], both groups performed a resistance training program; 1 group performed eccentric exercises, whereas the other concentric exercises (Table 2).

**Table 2:** Intervention characteristics and outcomes of the included studies.

Study	Exercise and intensity	Intervention protocol	Comparison	Strength measures	Outcomes / Main results	p^1^
Stegen et al., 2011	WU 10 min + Resistance 25 min (UL/LL, stack-weight, 2–3 sets of 15–10 rep, 60–75% 1RM)+ Aerobic 30 min (cycling, walking and stepping, 10 min each, 60–75% HR reserve) + CD 10 min	12 weeks, 3x/week, 75 min, Supervised	Usual Care	1RM (UL/LL); STS (30s); Handgrip	1RM Biceps(kg): IG 21.8±8.0 to 25.9±13.0 CG 27.3±9.6 to 20.8±8.8	**0.001**
					1RM Triceps(kg): IG 24.3±10.2 to 30.7±23.0 CG 30.1±10.5 to 22.0±6.6	**0.038**
					1RM Quadriceps(kg): IG 35.5±11.4 to 58.0±25.6 CG 57.3±28.2 to 45.9±25.1	**0.002**
					1RM Hamstrings(kg): IG 30.5±13.3 to 45.4±17.3 CG 39.0±35.0 to 35.3±27.8	0.094
					STS(rep): IG 13.0±3.0 to 16.0±4.0 CG 15.0±4.0 to 15.0±3.0	0.081
					Handgrip(kg): IG 76.5±30.4 to 67.6±20.2 CG 95.9±24.9 to 78.7±22.2	0.340
Huck, 2015	WU 10 min + Resistance 45 min (UL/LL, free/body/stack-weights/ resistive bands, 1–3 sets of 8–12 rep, 60–75% 1RM) + CD (stretching) 5 min	12 weeks, 2x/week (for 6 weeks), 3x/week (for 6 weeks), 60 min, Supervised	Usual Care Encouraged to increase daily PA and to consume protein shakes	1RM (UL/LL); 5-STS; Relative handgrip^3^	1RM Leg press(kg): IG 114.70±12.77 to 148.07±17.13 CG did not perform this test	**0.001** ^2^
					1RM Chest press(kg): IG 28.15±5.32 to 39.63±6.26 CG did not perform this test	**<0.001** ^2^
					Handgrip(kg): IG 1.09±0.30 to 1.21±0.24 CG 1.19±0.12 to 1.34±0.11	0.419
Campanha-Versiani et al., 2017	Resistance 60 min (UL/LL, weight-bearing, 1–3 sets of 10–12 rep, 10RM) + Aerobic 25 min (treadmill, 70–80% HRmax) PES was used to load evolution	36 weeks, 2x/week, 85 min, Supervised	Usual Care Instructed to maintain their usual PA and return for subsequent reevaluations	10RM (UL/LL)	10RM Ankle leg press(kg): IG 26.6±10.1 to 68.9±21.4 CG 26.6±18.9 to 45.8±19.5	**<0.01**
					10RM Seated leg curls(kg): IG 20.8±5.0 to 45.0±12 CG 20.8±14.0 to 32.6±12.3	**<0.01**
					10RM Bench press(kg): IG 13.9±6.3 to 30.0±14.6 CG 13.4±9.0 to 21.3±10.4	**<0.01**
					10RM Posterior shoulder(kg): IG 14.7±6.3 to 28.1±9.4 CG 11.6±6.9 to 13.7±6.6	**<0.01**
Coleman et al., 2017	Resistance (body weight, functional exercises) + Aerobic + Flexibility (dynamic balance and mobility) All exercises had intensity/difficulty levels and patients were allowed to increase the frequency and intensity of activities as they mastered lower levels of exercise	26 weeks, 2x/week (60 min structured exercise class), 3x/week (self-directed), Total: at least 150 min/week of moderate- to-vigorous PA, Semi-supervised	Usual Care Routine laboratory testing, weight assessment, and phone calls to encourage dietary changes and regular moderate-to-vigorous PA (2 weeks of surgery, at 2 and 6 months)	STS (30s); Arm curl	STS(rep): IG 11.0±3.4 to 11.6±4.2 CG 11.0±3.5 to 11.6±3.8	**>0.05**
					Arm curl(rep): IG 15.1±6.1 to 17.4±5.8 CG 15.0±3.8 to 16.2±4.0	**0.02**
Hassannejad et al., 2017	Aerobic (moderate intensity walking, 12–14 PES) OR Aerobic (moderate intensity walking, 12–14 PES) + Resistance 20–30 min (UL/LL, elastic bands)	12 weeks, 1–4 weeks (both groups): Aerobic 150min/week, 5–12 weeks: Aerobic: 3-5x/week 150-200min/week Resistance: 3x/week (+aerobic), Not Supervised	Usual Care No exercise was prescribed to the control group	1RM (UL); STS (60s)	1RM(kg) Resistance + Aerobic 17.7±9.4 to 18.7±9.0 CG 12.5±6.7 to 11.9±7.0	**0.004**
					1RM(kg) Aerobic 15.9±7.1 to 14.8±6.2 CG 12.5±6.7 to 11.9±7.0	0.348
					1RM(kg) Resistance + Aerobic: 17.7±9.4 to 18.7±9.0 Aerobic: 15.9±7.1 to 14.8±6.2	**0.031**
					STS(rep) Resistance + Aerobic 15.3±4.6 to 24.6±6.0 CG 7.8±5.3 to 17.0±6.2	0.142
					STS(rep) Aerobic 13.6±6.6 to 22.2±8.4 CG 7.8±5.3 to 17.0±6.2	0.267
					STS(rep) Resistance + Aerobic: 15.3±4.6 to 24.6±6.0 Aerobic: 13.6±6.6 to 22.2±8.4	0.608
Herring et al., 2017	WU 5 min + Resistance 10–20 min (LL, stack-weight/core, 3 sets of 12 rep, 60% 1RM) + Aerobic 35–45 min (64–77% HRmax / 12–14 PES) + CD	12 weeks, 3x/week, 60 min Supervised	Usual Care	Handgrip; 5-STS	Handgrip(kg): IG 27.6±8.7 to 29.99±7.9 CG 28.5±9.6 to 27.57±9.04	**0.036**
					STS(s): IG 13.7±6.8 to 9.9±3.7 CG 12.2±2.9 to 12.4±4.4	**0.010**
Daniels et al., 2018	WU and stretching 5–10 min + Resistance 50–60 min (UL/LL, core exercises and lifts, 1–4 sets of 8–15 rep, 50->80% 1RM) CD 5–10 min (stationary bicycle)	12 weeks, 3x/week, 60–80 min, NA if supervised or not	Usual Care Instructed to continue their normal daily activities during the 12-week study		1RM Leg press(kg): IG 163.4±34.4 to 222.8±42.4 CG 131.1±33.5 to 126.3±37.9	**<0.001**
					1RM Leg extension(kg): IG 32.5±6.0 to 38.3±6.4 CG 26.9±5.3 to 26.2±5.5	**0.014**
				1RM (LL); muscle quality^4^	Muscle quality (kg/cm2): IG 1.4±0.4 to 2.1±0.6 leg press CG 1.2±0.3 to 1.2±0.3	**<0.001**
					Muscle quality (kg/cm2): IG 0.62±0.22 to 0.74±0.27 leg extension CG 0.51±0.07 to 0.52±0.14	**<0.001**
Mundbjerg et al., 2018	Resistance 10 min (UL, free/stack-weights, 10–20 rep, 60–75% 1RM) + Aerobic 30 min (bike training + stair climbing OR treadmill walking OR rowing machine, PES and 50–70% VO2max) + Free access to the training facility and recommended a minimum of 3.5 h of PA per week (not supervised)	26 weeks, 2x/week, 40 min, Supervised	Usual Care Received standard information about the recommendation of PA post-surgery. There was no restriction on the amount of PA during the study period.	Isometric Dynamometer (LL/ UL); STS (30s)	Hip adduction(N): IG 145.2±36.2 to 153.5±40.0 CG 137.1±46.3 to 132.2±47.5	**0.007**
					Hip abduction(N): IG 141.7±36.6 to 147.1±34.9 CG 137.2±37.5 to 133.5±38.9	0.097
					Hip extension(N): IG 203.2±46.6 to 198.9±50.4 CG 198.7±60.0 to 196.0±58.8	0.678
					Shoulder adduction(N): IG 214.0±61.6 to 224.3±68.6 CG 196.9±67.1 to 193.3±77.5	0.199
					Shoulder abduction(N): IG 170.5±42.3 to 178.3±56.6 CG 160.1±54.9 to 166.1±65.9	0.889
					STS(rep): IG 15.0±3.5 to 16.1±3.2 CG 16.1±3.8 to 16.8±3.7	0.365
Kelley, 2019	Eccentric 5–30 min (LL, isokinetic machine, speed set at 23 rep/min, >138% 1RM) OR Concentric (LL, 70–80% 1RM, PES at least 7)	16 weeks, 3x/week 5–30 min Supervised	Usual care Counseling to obtain 150 minutes of moderate PA and 2 days of resistance training per week, regardless of group assignment.	1RM (LL); STS (30s); Isokinetic dynamometer (LL); Isometric dynamometer (LL)	1RM Leg press (kg): EcG 94.7±37.0 to 163.0±29.5 CcG 105.8±58.1 to 284.2±74.2 CG 107.6±39.3 to 123.5±48.3	**0.003**
					STS(rep): EcG 13.6±1.5 to 19.6±1.6 CcG 12.1±1.4 to 18.3±1.9 CG 13.3±1.5 to 17.6±1.6	0.67
					Knee extension (Nm): EcG 269.5±56.2 to 117.9±26.3 isokinetic CcG 242.4±54.5 to 146.5±22.0 CG 239.7±47.6 to 119.2±22.0	0.058
					Knee extension (Nm): EcG 297.3±71.2 to 138.6±26.3 isometric CcG 313.6±70.5 to 195.5±62.4 CG 247.5±75.6 to 149.8±33.0	0.198
Noack-Segovia et al., 2019	Resistance (UL, 1RM) + Aerobic (Treadmill 30 min, 54% of capacity and resistance frequency intensity, then modified to 59% of said frequency + cycle ergometer 15 min, without load); + CD (elongation/respiratory exercises)	24 weeks, 3x/week, 90 min Supervised	Usual care Monthly control by a physician and a nutritionist.	Handgrip	Handgrip(kg): IG 34.19±7.32 to 31.57±6.72 CG 34.18±8.91 to 31.91±9.12	>0.05
Gallé et al., 2020	WU 10 min + Aerobic 25 min (brisk walking 50–70% HRmax or 4 PES) + Resistance 15 min (UL/LL, 3 sets of 12 rep, 70–85% 1RM) + CD 5 min (agility/balance) + Flexibility 5 min (static/dynamic)	54 weeks, 2x/week, 60 min Supervised	Usual Care Meeting with bariatric surgeon at 1 and 12 months after surgery, and counseling regarding diet and PA. Possibility to see a dietitian upon request	STS (rep until exhaustion); Handgrip	Handgrip(kg): IG 32.9±10.5 to 49.2±15.1 CG 32.8±11.9 to 34.0±11.6	**<0.01**
					STS(rep): IG 51.8±21.2 to 98.8±26.5 CG 49.2±19.1 to 47.9±13.6	**<0.01**
de Oliveira Junior et al., 2021	WU 5 min + Resistance ≈60 min (UL/LL, 4–5 sets of 10–15 rep, 6–8 PES, home-based) + Aerobic 30–50 min (walking/running/stair climbing, 10 min progression every 4 weeks, 6–8 PES)	12 weeks 3x/week ≈90–110 min Semi-supervised	Usual care	Handgrip; STS (30s)	Handgrip(kg): IG 35.4±9.6 to 34.0±8.2 CG 35.3±11.0 to 33.9±11.2	**0.99**
					STS(rep): IG 12.8±2.2 to 15.0±2.6 CG 13.4±2.5 to 13.8±2.5	**<0.01**
Diniz-Souza et al., 2021	WU 5 min + High impact 20 min (high ground-reaction force exercises, 183 to 209 gravitational loading peaks above 4.9 g) + Balance 10 min (static/dynamic) + Resistance 35 min (UL/LL, 2–3 sets of 4–12 rep, 65–85% 1RM) + CD 5 min	48 weeks, 3x/week, 75 min Supervised	Usual care Verbal recommendation to increase PA	Isokinetic dynamometer (LL)	Knee extension: IG 156.19±36.85 to 133.76±35.45 (Nm) CG 156.31±32.71 to 130.39±32.02	>0.05
					Knee flexion: IG 79.69±18.42 to 76.04±17.48 (Nm) CG 80.55±16.57 to 69.73±16.07	>0.05
Gil et al., 2021	WU 5 min + Resistance ≈40 min (UL/LL, 3 sets of 8–12 rep, 5% load progression when ≥ 2 rep were performed than previously determined) + Aerobic 30–60 min (treadmill, 10 min progression every 4 weeks, 50% of the delta difference between the ventilatory anaerobic threshold and respiratory compensation point)	24 weeks, 3x/week 70–100 min Supervised	Usual care	1RM (UL/LL); STS (30s)	1RM Bench press (kg): IG 26.31±7.28 to 31.07±7.55 CG 26.87±7.25 to 24.77±5.57	**<0.0001**
					1RM Leg press (kg): IG 148.77±50.50 to 214.63±59.79 CG 130.36±49.03 to 120.37±47.40	**<0.0001**
					STS(rep): IG 14.0±2.0 to 18.0±3.0 CG 13.0±2.0 to 14.0±1.0	**<0.0001**
Lamarca et al., 2021	WU 10 min + Resistance 40 min (UL/LL, stack/free weights, 3 sets of 8–12 reps, 6–8 OMNI-RES) + CD 10 min (elongation/respiratory exercises)	12 weeks, 3x/week, 60 min Supervised	Usual care All participants received general training on healthy eating	Isokinetic dynamometer (LL); STS(30s)	Knee extension(Nm): IG 153.2±52.1 to 164.8±54.4 CG 124.2±23.8 to 122.3±23.0	**<0.05**
					STS(rep): IG 14.75±2.0 to 17.64±2.33 CG 15.29±2.47 to 16.0±3.14	>0.05

CcG, Concentric group; CD, Cooldown; CG, Control group; CM, centimeter; EcG, Eccentric group; HR, Heart rate; IG, Intervention group; LL, Lower limb; Max, Maximum; Min, Minutes; N, Newton; NA, Not available; OMNI-RES, OMNI-Resistance Exercise Scale; PA, Physical Activity; PES, Borg’s Perceived Exertion Scale; Rep, repetitions; RM, Repetition maximum; S, Seconds; STS, Sit-to-stand test; UL, Upper limb; VO_2_, volume of oxygen; WU, Warmup

^1^P value compared to the control group;

^2^ P value compared to baseline;

^3^ (Right hand + left hand)/fat-free mass;

^4^Muscle quality = 1RM muscle strength/muscle cross-sectional of the right tight (kg/cm^2^);

Training sessions mainly lasted from 30 [36] to 90 minutes [6, 37, 41] and were performed 2 [29–31, 35, 38] to 5 times weekly [31, 32] over 12 [6, 28, 29, 32–34, 40, 41] to 54 weeks [38]. The training was supervised by exercise specialists in most studies [6, 28–30, 33, 35–40].

The types of aerobic training most frequently performed were walking [6, 28, 30, 32, 35, 37, 38, 41], cycling [28, 35, 37], and stepping/stair climbing [28, 35, 41]. Two studies did not describe the method of aerobic training [35, 37]. To ensure the intensity of aerobic training, most studies used Borg’s Perceived Exertion Scale [32, 33, 35, 41] and monitored the heart rate [30, 38].

In terms of resistance training, most studies involved both lower and upper limbs [6, 28–30, 32, 34, 38–41], whereas 2 studies involved only the upper limbs [35, 37], and 2 other studies involved only the lower limbs [33, 36]. Coleman et al. [31] did not specify the muscle groups involved. A percentage of one repetition maximum (RM) test [28, 29, 33–39] and Borg’s Perceived Exertion Scale [32, 36, 41] were most commonly used to verify the intensity level.

The control group received usual care after BS in most studies [6, 28, 30, 32–34, 37, 38, 40, 41]. However, some studies also encouraged patients in the control group to increase their physical activity level [29, 31, 36, 39]. Mundbjerg et al. [35] did not establish restrictions on physical activity in the control group.

### Synthesis of results and certainty of the evidence

To estimate MS, the studies used the 1RM [6, 28, 29, 32, 34, 36], 10RM [30], handgrip [28, 29, 33, 37, 38, 41], or dynamometer tests, while applying the isokinetic [39, 40], isometric [35], or both [36] protocols. Most of the studies performed the sit-to-stand test [6, 28, 29, 31–33, 35, 36, 38, 40, 41] for 30 seconds [6, 28, 31, 35, 36, 40, 41].

**Fig 3:**
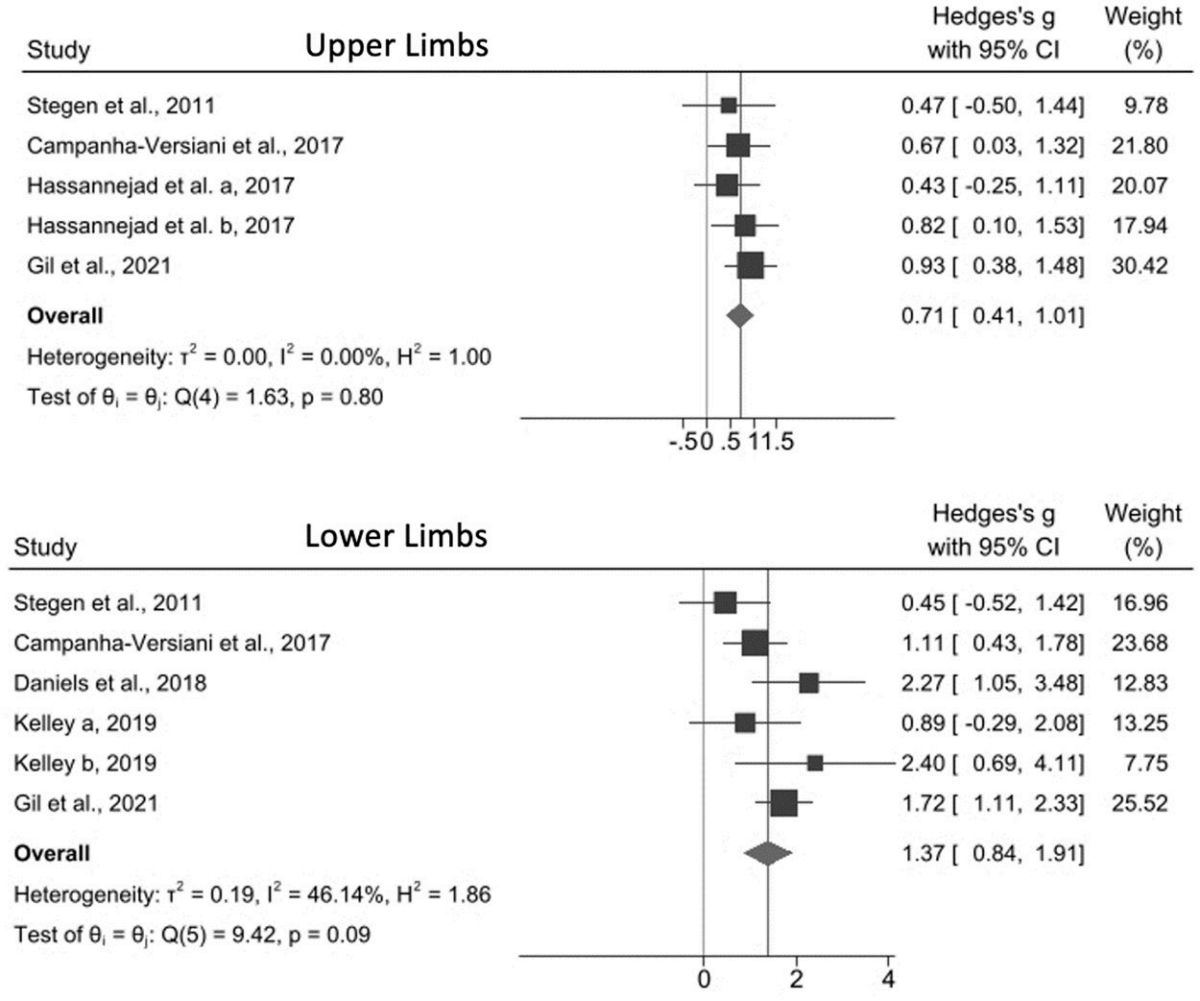
Effect size of physical exercise on muscle strength in adults following bariatric surgery according to the repetition maximum test. Hassanejad et al. a, 2017: aerobic training; Hassanejad et al. b, 2017: aerobic and resistance training; Kelley a, 2019: resistance eccentric; Kelley b, 2019: resistance concentric.

**Fig 4:**
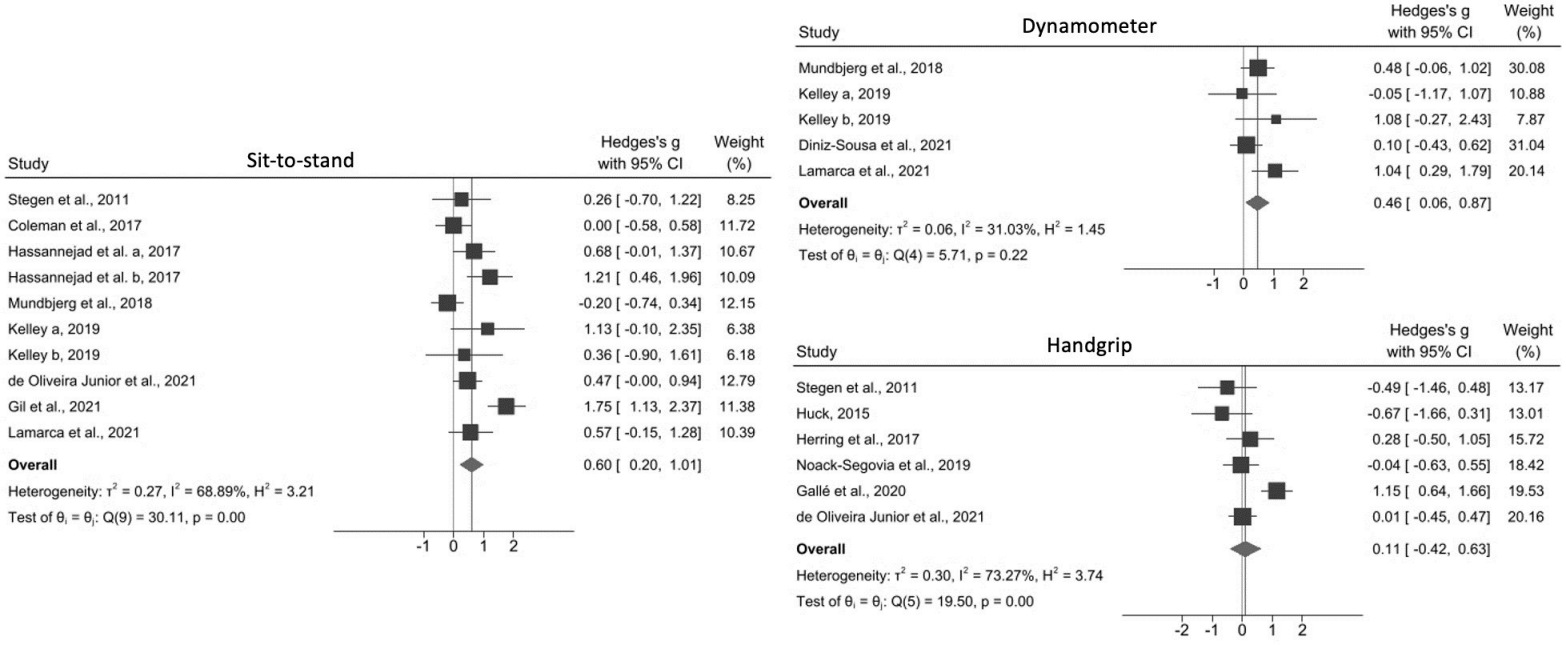
Effect size of physical exercise on muscle strength in adults following bariatric surgery according to the sit-to-stand, dynamometer, and handgrip tests. Hassanejad et al. a, 2017: aerobic training; Hassanejad et al. b, 2017: aerobic and resistance training; Kelley a, 2019: resistance eccentric; Kelley b, 2019: resistance concentric.

With the exception of Noack-Segovia et al. [37] and Diniz-Souza et al. [39], all studies demonstrated that exercise positively affected MS when evaluated by at least 1 assessment test (Table 2). The meta-analyses showed that exercise interventions improved both upper (effect size, 0.71; 95% CI, 0.41–1.01; I^2^ = 0%) and lower limb MS (effect size, 1.37; 95% CI, 0.84–1.91; I^2^ = 46.14) when RM tests were used (Fig 3). Similar results were obtained with the sit-to-stand (effect size, 0.60; 95% CI, 0.20–1.01; I^2^ = 68.89%) and dynamometer (effect size, 0.46; 95% CI, 0.06–0.87; I^2^ = 31.03%) tests but not with the handgrip test (effect size, 0.11; 95% CI, 0.42–0.63; I^2^ = 73.27%) (Fig 4).

Herring et al. [33] was excluded from the meta-analysis for the sit-to-stand test as they used a different test methodology. Galle et al.’s study [38] was also excluded because of a high level of heterogeneity that was attributed to the lack of a 30- or 60-second time limit and the performance of tests until exhaustion.

The 5 meta-analyses yielded a very low certainty of evidence according to the GRADE evaluation (S3 Table). None of the individual studies had a low risk of bias. Therefore, the included studies contributed more than 50% of the weight to the pooled estimate for each meta-analysis. For inconsistency, 2 meta-analyses demonstrated highly significant heterogeneity, whereas another 1 showed moderate non-significant heterogeneity. Regarding indirectness, all meta-analyses were downgraded 1 level due to a high degree of variability in the exercise protocols; 2 meta-analyses were affected by population heterogeneity, particularly concerning postoperative time. Regarding imprecision, none of the meta-analyses included the minimum sample size of 400 patients. Despite the estimate of treatment effect favoring the intervention in the handgrip meta-analysis, the 95% CI included the null value (S3 Table).

As none of the meta-analyses included more than 10 studies, Egger’s test could not be used to assess publication bias. Therefore, we assessed publication bias by evaluating the search strategy and use of industry funding; the results indicated that none of the meta-analyses were affected by publication bias.

## Discussion

Current evidence indicates that physical exercise interventions, especially with a resistance training component, may be effective in increasing MS in patients following BS [6, 28–36, 38, 40, 41]. Analysis of MS by the RM test showed that physical exercise was effective for both the upper and lower limbs. Similar results were found with the sit-to-stand and dynamometer tests but not with the handgrip test. Notably, all studies included in this systematic review were not appraised as having a low risk of bias, and the results of all 5 meta-analyses had very low levels of certainty. Despite the moderate effects, our results need to be considered in the context of the negative impact of BS on FFM and MS, with elevated risk for sarcopenic obesity.

Our findings are consistent with those of previous systematic reviews [10, 11, 18, 19]. Nevertheless, we accounted for the use of different MS assessment methods, which focus on different muscle groups and types of strength. Additionally, our review included several recent studies that have not been incorporated in prior meta-analyses.

The general population is recommended to participate regularly in resistance training to increase MS. However, there are currently no specific guidelines for physical activity or exercise in individuals following BS, and existing training protocols vary widely in type, intensity, duration, and frequency [8].

A large national cohort study showed that obesity, low MS, and low aerobic fitness were independently associated with increased mortality [42], and even small changes in either upper or lower limb MS can affect the mortality risk [17]. Moreover, MS and aerobic fitness had interactive effects, thus demonstrating the need to promote both dimensions of physical fitness, especially for individuals with obesity [42]. The combination of resistance training with aerobic exercise, when compared with isolated aerobic exercise, was superior regarding weight loss, functional capacity, FFM, and MS after BS [32, 43].

The following factors must be considered when assessing MS: muscle contraction type, measurement system, test equipment, pattern and range of motion, and loading scheme [44]. Isokinetic dynamometers are commonly used for MS assessment in the laboratory for the validation of other strength assessment measurements [45] and are used to evaluate isometric and isokinetic peak torque [46]. However, they are expensive and generally only evaluate a single-joint muscle exercise; furthermore, the movement performed does not resemble that used in routine activities [47].

1RM and isometric tests are generally used for MS assessment in clinical settings. The 1RM is defined as the maximum weight that can be lifted once while maintaining the correct lifting technique [48]. The 1RM test has some advantages, such as allowing the evaluation of multi-joint exercises making it better able to reflect dynamic muscle actions that are used in daily life; it is also widely used and cost-effective. However, populational studies can be time-consuming [49]. 1RM test reliability tends to be excellent, regardless of age, sex, body part assessed, and experience in resistance training [50]. The 1RM can also be predicted through 5–10 submaximal repetitions by equations that are exercise and population specific, which do not submit individuals to their maximum external loads; however, tests with more than 10 repetitions are not recommended [51].

Isometric strength tests, such as the handgrip test, are versatile, time-efficient, and strongly correlated with maximum dynamic strength during similar movement patterns [46, 52]. However, they require specialized devices such as a tension gauge or force platform [44]. In this systematic review, the handgrip test was unable to detect the positive effects of exercise on MS in cases where effects could be detected by other assessment tests [28, 29, 41]. The sensitivity of a MS assessment test may be specific to the training program performed [44]. Exercise interventions with a resistance training component that included manual isometric exercises were able to increase MS measured with handgrip test in different clinical populations [43, 53, 54].

The sit-to-stand test assesses an individual’s ability to independently get up from a chair. It has a good correlation with lower limb MS and the 6-minute walk test and is commonly used in the elderly, healthy young adults, and clinical populations [55–57]. Special attention is required when interpreting the results of the sit-to-stand test owing to methodological variations in the maximum number of repetitions performed within a 30- or 60-second time interval [58] and the time required to perform a predetermined number of repetitions (e.g., 5–10) [59].

This review has some limitations. First, our data were limited to a small number of clinical trials (with restrictive sample sizes), which limits the random-effects model interpretation. Second, none of the included studies had a low risk of bias, and all results generated by the meta-analyses had very low levels of certainty. Third, there was a high level of heterogeneity among the included studies due to differences in interventions. Thus, we were unable to assess the effect of various study characteristics on the observed estimates. Fourth, most of the studies focused on the early postoperative period, during which there is a large loss of weight, FFM, and absolute MS. Lastly, for the lower limbs’ dynamometer meta-analysis, isokinetic and isometric data were pooled in the same analysis, due to limited number of studies, which did not allow separated investigations. However, even though they represent two different aspects of strength production [20], they are highly correlated [60, 61], and were performed in similar devices.

The strengths of this review include the protocol registration in PROSPERO, a wide independently literature search following the PRESS recommendations, and the manual check of the reference lists. To ensure transparency of reporting, we adhered to the 2020 PRISMA guide [21], Cochrane handbook for performing meta-analyses [25], and GRADE [27] approach. Furthermore, we included trials with a wide range of characteristics to increase the generalizability of our results. To our knowledge, this is the first meta-analysis to evaluate the effect of exercise on MS assessed with different methodologies in individuals following BS.

In conclusion, physical exercise with a resistance training component performed after BS may improve MS, a variable closely related to sarcopenic obesity, functional disability and mortality risk, therefore it is essential to be performed as an adjuvant therapy in the postoperative follow-up care. Improvements in MS were observed when assessments were made with the RM (upper and lower limbs), sit-to-stand, and dynamometer tests, but not with handgrip test. Knowing in depth the MS assessment methods most used in research and in clinical practice helps the practitioner to choose the most appropriate method for the target population and purposes. Additional high-quality randomized clinical trials are required to determine the optimal exercise intervention protocol to improve MS for this population.

## Supporting information

S1 Checklist Preferred reporting items for systematic reviews and meta-analyses (PRISMA) checklist. (DOCX)

S1 File International Prospective Register of Systematic Reviews (PROSPERO)—CRD42020152142. (PDF)

S1 Table Database search strategy. (DOCX)

S2 Table Risk of bias for each individual study assessed by Joanna Briggs Institute checklist for randomized controlled trials. (DOCX)

S3 Table Summary of findings for certainty of the evidence (GRADE). (DOCX)
