# Supplementary material for: Effect of physical exercise on muscle strength in adults following bariatric surgery: A systematic review and meta-analysis of different muscle strength assessment tests
Source: PLoS One. 2022 Jun 10;17(6):e0269699. doi: 10.1371/journal.pone.0269699 (PMC9187088; doi:10.1371/journal.pone.0269699)
Supplement: S1 File — (PDF) [file pone.0269699.s002.pdf]

To enable PROSPERO to focus on COVID-19 submissions, this registration record has undergone basic automated checks for eligibility and is published exactly as submitted. PROSPERO has never provided peer review, and usual checking by the PROSPERO team does not endorse content. Therefore, automatically published records should be treated as any other PROSPERO registration. Further detail is provided here.

Review methods were amended after registration. Please see the revision notes and previous versions for detail.

Citation

Flavio Vieira, Gabriela de Oliveira, Vivian Gonçalves, Silvia Neri, Kenia de Carvalho, Eliane Dutra. Effect of physical exercise on strength and muscle mass of adults who underwent bariatric surgery: a systematic review. PROSPERO 2020 CRD42020152142 Available from:
https://www.crd.york.ac.uk/prospero/display_record.php?ID=CRD42020152142

Review question [1 change]

Does resistance and/or aerobic physical training promote increase in strength and/or muscle mass in adults in the postoperative period of bariatric surgery?

Searches [1 change]

EMBASE, MEDLINE, LILACS, Web of Science, Scopus, SPORTDiscus, ProQuest, Google Scholar.

Search date: October 1st

Inclusion criteria

Adults

Male and female

Any postoperative time of bariatric surgery (Roux-en-Y gastric bypass or Sleeve gastrectomy)

Studies containing information about type, frequency and duration of exercise

Strength or muscle mass and/or fat free mass data, evaluated by any method

No language and publication period restrictions

Exclusion criteria

Studies that exclusively evaluate specific populations with chronic diseases

Studies that have supplementation of ergogenic resources associated with exercise

Unpublished studies will not be sought for this review

Searches will be re-run prior to final analysis

Types of study to be included [1 change]

Inclusion criteria: Clinical trials and observational studies

Exlusion criteria: Letters, review articles, personal opinions, book chapters, or conference summaries.

Condition or domain being studied
Bariatric surgery is an effective procedure for weight loss and remission of comorbidities associated with severe obesity. On the other hand, patients who underwent this surgical method may have a substantial reduction in muscle mass, or may have a consequent impact on activity performance capacity and absolute muscle strength. The practice of regular physical exercises might favor the body composition and the muscle strength of these patients.

Participants/population
Adults (over 18 and under 70 years old) who underwent bariatric surgery (Roux-en-Y Gastric Bypass or Sleeve gastrectomy) at any postoperative time, male and female.

Intervention(s), exposure(s)
Resistance and/or aerobic physical exercise

Comparator(s)/control [1 change]

Adults who underwent Roux-en-Y Gastric Bypass or sleeve gastrectomy and are not engaged in a physical exercise program

Main outcome(s) [1 change]

Strength and muscle mass, measured by any method

Measures of effect

Changes in strength and muscle mass from baseline to the last available follow-up, measured by any method

Additional outcome(s)
Body composition, measured by any method

Measures of effect

Changes in body composition from baseline to the last available follow-up, measured by any method

Data extraction (selection and coding)
The selection process will be done in two phases. In phase 1, two reviewers independently will read the titles and abstracts of all identified electronic database citations (FTV; GOS). Any studies that do not fulfill the inclusion criteria

will be discarded. In phase 2, the same selection criteria will be applied to the full articles to confirm their eligibility.

The three reviewers (FTV; GOS; RML) will participate independently in phase 2. The references list of included

articles will be revised by two main reviewers (FTV; GOS). Any disagreement will be resolved by a consensus. If

they not reach a consensus, the coordinator will make a final decision (ESD). Final selection will be always based

on the full-text of the publication and afterwards.

Risk of bias (quality) assessment
The critical appraisal checklist for randomized controlled trials by The Joanna Briggs Institute (JBI) will be used to assess the risk of bias of included studies. Two reviewers (FTV and GOS) will assess independently the quality of each included study. Disagreements will be solved by consensus or by the decision of a third reviewer (RML). The results of the risk of bias analysis and the characteristics of methodological quality,

such as randomization, blinding and statistical analysis, will be present in narrative text and in tables. The JBI Critical Appraisal Checklist for Randomized Controlled Trials consists of thirteen questions that will be answered as “yes”, “no”, “unclear”, or “not applicable”, enabling assessment of the studies as having a high or low risk of bias according to the answers.

Strategy for data synthesis [1 change]

For the included studies the following information will be recorded: author, year of publication, country, study design, sample characteristics and number of participants, type/frequency/duration of the exercise, main outcomes: strength and muscle mass, and method used for evaluation.

Analysis of subgroups or subsets

None planned.

Contact details for further information

Flavio Vieira
flavio.nut@hotmail.com

Organisational affiliation of the review

University of Brasilia
www.unb.br

Review team members and their organisational affiliations [1 change]

Mr Flavio Vieira. University of Brasilia
Miss Gabriela de Oliveira. University of Brasilia
Dr Vivian Gonçalves. University of Brasilia
Dr Silvia Neri. University of Brasilia
Dr Kenia de Carvalho. University of Brasilia
Dr Eliane Dutra. University of Brasilia

Type and method of review

Intervention, Meta-analysis, Systematic review

Anticipated or actual start date [1 change]

01 March 2021

Anticipated completion date [1 change]

05 April 2022

Funding sources/sponsors

None

Conflicts of interest

None specified
None known

Language

English

Country

Brazil

Stage of review [1 change]

Review Completed not published

Subject index terms status
Subject indexing assigned by CRD

Subject index terms
MeSH headings have not been applied to this record

Date of registration in PROSPERO
28 April 2020

Date of first submission
27 September 2019

Details of any existing review of the same topic by the same authors
None

Stage of review at time of this submission [1 change]

Stage	Started	Completed
Preliminary searches	Yes	Yes
Piloting of the study selection process	Yes	Yes
Formal screening of search results against eligibility criteria	Yes	Yes
Data extraction	Yes	Yes
Risk of bias (quality) assessment	Yes	Yes
Data analysis	Yes	Yes

Revision note
We decided not to report findings for muscle mass, since other systematic reviews with meta-analysis have already been published of this theme in the meantime

The record owner confirms that the information they have supplied for this submission is accurate and complete and they understand that deliberate provision of inaccurate information or omission of data may be construed as scientific misconduct.
The record owner confirms that they will update the status of the review when it is completed and will add publication details in due course.

Versions
28 April 2020
04 April 2022
