# Supplementary material for: Effect of physical exercise on muscle strength in adults following bariatric surgery: A systematic review and meta-analysis of different muscle strength assessment tests
Source: PLoS One. 2022 Jun 10;17(6):e0269699. doi: 10.1371/journal.pone.0269699 (PMC9187088; doi:10.1371/journal.pone.0269699)
Supplement: S1 Table — (DOCX) [file pone.0269699.s003.docx]

| **DATABASE** | **SEARCH** (27th October 2021) |
| --- | --- |
| **EMBASE** | ('bariatric surgery' OR 'bariatric surgeries' OR 'obesity surgery' OR 'metabolic surgery' OR 'metabolic surgeries' OR 'bariatric surgical procedures' OR 'bariatric surgical procedure' OR 'gastric bypass' OR 'roux-en-y gastric bypass' OR gastroplasty OR 'roux en y gastric bypass' OR sleeve OR 'sleeve gastrectomy' OR 'vertical sleeve') AND ('physical activity' OR 'physical activities' OR 'physical fitness' OR 'physical training' OR exercise OR 'physical exercise' OR 'physical exercises' OR 'exercise training' OR 'exercise trainings' OR 'exercise test' OR 'exercise tests' OR 'physical fitness testing' OR 'fitness testing' OR 'aerobic exercise' OR 'aerobic exercises' OR 'aerobic training program' OR 'exercise therapy' OR 'exercise movement technics' OR 'endurance training' OR 'physical endurance' OR 'performance task' OR 'performance tasks' OR 'performances task' OR 'task performance analysis' OR 'task performances analysis' OR 'resistance training' OR 'resistance training intervention' OR 'resistance training program' OR 'strength training' OR 'weight-lifting strengthening program' OR 'weight-lifting strengthening programs' OR 'weight-lifting exercise program' OR 'weight-lifting exercise programs' OR 'weight-bearing strengthening program' OR 'weight-bearing strengthening programs' OR 'weight-bearing exercise program' OR 'weight-bearing exercise programs') AND (strength OR torque OR 'peak torque' OR 'muscle strength' OR 'force production' OR 'physical function' OR 'hand strengths' OR 'hand strength' OR 'muscle contraction' OR 'muscle contractions' OR 'muscular contraction' OR 'muscular contractions' OR 'lean body mass' OR 'muscle mass' OR 'fat free mass' OR 'muscle cross-sectional area' OR 'muscle cross sectional area' OR 'body composition' OR 'body compositions' OR 'body constitution' OR 'body constitutions' OR anthropometry OR 'body weights measures' OR 'body measures' OR 'body size' OR 'muscle fatigue' OR 'muscle protein' OR 'muscle proteins' OR 'skeletal muscle' OR 'skeletal muscles' OR 'skeletal muscle mass' OR 'muscle thickness' OR hypertrophy OR hyperplasia) |
| **MEDLINE** | ("Bariatric surgery"[All Fields] OR "Bariatric surgeries"[All Fields] OR "Obesity surgery"[All Fields] OR "Metabolic surgery"[All Fields] OR "Metabolic surgeries"[All Fields] OR "Bariatric surgical procedures"[All Fields] OR "Bariatric surgical procedure"[All Fields] OR "Gastric bypass"[All Fields] OR "roux en y gastric bypass"[All Fields] OR ("gastroplasty"[MeSH Terms] OR "gastroplasty"[All Fields] OR "gastroplasties"[All Fields]) OR "roux en y gastric bypass"[All Fields] OR ("sleeve"[All Fields] OR "sleeved"[All Fields] OR "sleeves"[All Fields] OR "sleeving"[All Fields]) OR "Sleeve gastrectomy"[All Fields] OR "Vertical sleeve"[All Fields]) AND ("Physical activity"[All Fields] OR "Physical activities"[All Fields] OR "Physical fitness"[All Fields] OR "Physical training"[All Fields] OR ("exercise"[MeSH Terms] OR "exercise"[All Fields] OR "exercises"[All Fields] OR "Exercise therapy"[MeSH Terms] OR ("exercise"[All Fields] AND "therapy"[All Fields]) OR "Exercise therapy"[All Fields] OR "exercise s"[All Fields] OR "exercised"[All Fields] OR "exerciser"[All Fields] OR "exercisers"[All Fields] OR "exercising"[All Fields]) OR "Physical exercise"[All Fields] OR "Physical exercises"[All Fields] OR "Exercise training"[All Fields] OR "Exercise trainings"[All Fields] OR "Exercise test"[All Fields] OR "Exercise tests"[All Fields] OR "Physical fitness testing"[All Fields] OR "Fitness testing"[All Fields] OR "Aerobic exercise"[All Fields] OR "Aerobic exercises"[All Fields] OR "Aerobic training program"[All Fields] OR "Exercise therapy"[All Fields] OR "Exercise movement technics"[All Fields] OR "Endurance training"[All Fields] OR "Physical endurance"[All Fields] OR "Performance task"[All Fields] OR "Performance tasks"[All Fields] OR ("task performance and analysis"[MeSH Terms] OR ("task"[All Fields] AND "performance"[All Fields] AND "analysis"[All Fields]) OR "task performance and analysis"[All Fields] OR ("performances"[All Fields] AND "task"[All Fields])) OR "Task performance analysis"[All Fields] OR ("task performance and analysis"[MeSH Terms] OR ("task"[All Fields] AND "performance"[All Fields] AND "analysis"[All Fields]) OR "task performance and analysis"[All Fields] OR ("task"[All Fields] AND "performances"[All Fields] AND "analysis"[All Fields])) OR "Resistance training"[All Fields] OR "Resistance training intervention"[All Fields] OR "Resistance training program"[All Fields] OR "Strength training"[All Fields] OR ("Resistance training"[MeSH Terms] OR ("resistance"[All Fields] AND "training"[All Fields]) OR "Resistance training"[All Fields] OR ("weight"[All Fields] AND "lifting"[All Fields] AND "strengthening"[All Fields] AND "program"[All Fields])) OR ("Resistance training"[MeSH Terms] OR ("resistance"[All Fields] AND "training"[All Fields]) OR "Resistance training"[All Fields] OR ("weight"[All Fields] AND "lifting"[All Fields] AND "strengthening"[All Fields] AND "programs"[All Fields])) OR "Weight-lifting exercise program"[All Fields] OR "Weight-lifting exercise programs"[All Fields] OR "Weight-bearing strengthening program"[All Fields] OR "Weight-bearing strengthening programs"[All Fields] OR "Weight-bearing exercise program"[All Fields] OR "Weight-bearing exercise programs"[All Fields]) AND ("strength"[All Fields] OR "strengths"[All Fields] OR ("torque"[MeSH Terms] OR "torque"[All Fields] OR "torques"[All Fields] OR "torqued"[All Fields] OR "torqueing"[All Fields] OR "torquing"[All Fields]) OR "peak torque"[All Fields] OR "Muscle strength"[All Fields] OR "Force production"[All Fields] OR "Physical function"[All Fields] OR "Hand strengths"[All Fields] OR "Hand strength"[All Fields] OR "Muscle contraction"[All Fields] OR "Muscle contractions"[All Fields] OR "Muscular contraction"[All Fields] OR "Muscular contractions"[All Fields] OR "Lean body mass"[All Fields] OR "Muscle mass"[All Fields] OR "Fat free mass"[All Fields] OR "muscle cross sectional area"[All Fields] OR "muscle cross sectional area"[All Fields] OR "Body composition"[All Fields] OR "Body compositions"[All Fields] OR "Body constitution"[All Fields] OR "Body constitutions"[All Fields] OR ("anthropometries"[All Fields] OR "anthropometry"[MeSH Terms] OR "anthropometry"[All Fields]) OR (("body weight"[MeSH Terms] OR ("body"[All Fields] AND "weight"[All Fields]) OR "body weight"[All Fields] OR ("body"[All Fields] AND "weights"[All Fields]) OR "body weights"[All Fields]) AND ("measurability"[All Fields] OR "measurable"[All Fields] OR "measurably"[All Fields] OR "measure s"[All Fields] OR "measureable"[All Fields] OR "measured"[All Fields] OR "measurement"[All Fields] OR "measurement s"[All Fields] OR "measurements"[All Fields] OR "measurer"[All Fields] OR "measurers"[All Fields] OR "measuring"[All Fields] OR "measurings"[All Fields] OR "measurment"[All Fields] OR "measurments"[All Fields] OR "weights and measures"[MeSH Terms] OR ("weights"[All Fields] AND "measures"[All Fields]) OR "weights and measures"[All Fields] OR "measure"[All Fields] OR "measures"[All Fields])) OR "Body measures"[All Fields] OR "Body size"[All Fields] OR "Muscle fatigue"[All Fields] OR "Muscle protein"[All Fields] OR "Muscle proteins"[All Fields] OR "Skeletal muscle"[All Fields] OR "Skeletal muscles"[All Fields] OR "Skeletal muscle mass"[All Fields] OR "Muscle thickness"[All Fields] OR ("hypertrophy"[MeSH Terms] OR "hypertrophy"[All Fields] OR "hypertrophied"[All Fields] OR "hypertrophies"[All Fields] OR "hypertrophying"[All Fields]) OR ("hyperplasia"[MeSH Terms] OR "hyperplasia"[All Fields] OR "hyperplasias"[All Fields])) |
| **SCOPUS** | TITLE-ABS-KEY ( "Bariatric surgery" OR "Bariatric surgeries" OR "Obesity surgery" OR "Metabolic surgery" OR "Metabolic surgeries" OR "Bariatric surgical procedures" OR "Bariatric surgical procedure" OR "Gastric bypass" OR "Roux-en-Y gastric bypass" OR gastroplasty OR "Roux en Y Gastric Bypass" OR sleeve OR "Sleeve gastrectomy" OR "Vertical sleeve" ) AND TITLE-ABS-KEY ( "Physical activity" OR "Physical activities" OR "Physical fitness" OR "Physical training" OR exercise OR "Physical exercise" OR "Physical exercises" OR "Exercise training" OR "Exercise trainings" OR "Exercise test" OR "Exercise tests" OR "Physical fitness testing" OR "Fitness testing" OR "Aerobic exercise" OR "Aerobic exercises" OR "Aerobic training program" OR "Exercise therapy" OR "Exercise movement technics" OR "Endurance training" OR "Physical endurance" OR "Performance task" OR "Performance tasks" OR "Performances task" OR "Task performance analysis" OR "Task Performances Analysis" OR "Resistance training" OR "Resistance training intervention" OR "Resistance training program" OR "Strength training" OR "Weight-lifting strengthening program" OR "Weight-lifting strengthening programs" OR "Weight-lifting exercise program" OR "Weight-lifting exercise programs" OR "Weight-bearing strengthening program" OR "Weight-bearing strengthening programs" OR "Weight-bearing exercise program" OR "Weight-bearing exercise programs" ) AND TITLE-ABS-KEY ( strength OR torque OR "peak torque" OR "Muscle strength" OR "Force production" OR "Physical function" OR "Hand strengths" OR "Hand strength" OR "Muscle contraction" OR "Muscle contractions" OR "Muscular contraction" OR "Muscular contractions" OR "Lean body mass" OR "Muscle mass" OR "Fat free mass" OR "Muscle cross-sectional area" OR "Muscle cross sectional area" OR "Body composition" OR "Body compositions" OR "Body constitution" OR "Body constitutions" OR anthropometry OR "Body weights Measures" OR "Body measures" OR "Body size" OR "Muscle fatigue" OR "Muscle protein" OR "Muscle proteins" OR "Skeletal muscle" OR "Skeletal muscles" OR "Skeletal muscle mass" OR "Muscle thickness" OR hypertrophy OR hyperplasia ) |
| **SPORTDISCUS** | ( “Bariatric surgery” OR “Bariatric surgeries” OR “Obesity surgery” OR “Metabolic surgery” OR “Metabolic surgeries” OR “Bariatric surgical procedures” OR “Bariatric surgical procedure” OR “Gastric bypass” OR “Roux-en-Y gastric bypass” OR Gastroplasty OR “Roux en Y Gastric Bypass” OR Sleeve OR “Sleeve gastrectomy” OR “Vertical sleeve” ) AND ( “Physical activity” OR “Physical activities” OR “Physical fitness” OR “Physical training” OR Exercise OR “Physical exercise” OR “Physical exercises” OR “Exercise training” OR “Exercise trainings” OR “Exercise test” OR “Exercise tests” OR “Physical fitness testing” OR “Fitness testing” OR “Aerobic exercise” OR “Aerobic exercises” OR “Aerobic training program” OR “Exercise therapy” OR “Exercise movement technics” OR “Endurance training” OR “Physical endurance” OR “Performance task” OR “Performance tasks“ OR “Performances task” OR “Task performance analysis” OR “Task Performances Analysis” OR “Resistance training” OR “Resistance training intervention” OR “Resistance training program” OR “Strength training” OR “Weight-lifting strengthening program” OR “Weight-lifting strengthening programs” OR “Weight-lifting exercise program” OR “Weight-lifting exercise programs” OR “Weight-bearing strengthening program” OR “Weight-bearing strengthening programs” OR “Weight-bearing exercise program” OR “Weight-bearing exercise programs” ) AND ( Strength OR Torque OR “peak torque” OR “Muscle strength” OR “Force production” OR “Physical function” OR “Hand strengths” OR “Hand strength” OR “Muscle contraction” OR “Muscle contractions” OR “Muscular contraction” OR “Muscular contractions” OR “Lean body mass” OR “Muscle mass” OR “Fat free mass” OR “Muscle cross-sectional area” OR “Muscle cross sectional area” OR “Body composition” OR “Body compositions” OR “Body constitution” OR “Body constitutions” OR Anthropometry OR “Body weights Measures” OR “Body measures” OR “Body size” OR “Muscle fatigue” OR “Muscle protein” OR “Muscle proteins” OR “Skeletal muscle” OR “Skeletal muscles” OR “Skeletal muscle mass” OR “Muscle thickness” OR Hypertrophy OR hyperplasia ) |
| **WEB OF SCIENCE** | ((ALL=("Bariatric surgery" OR "Bariatric surgeries" OR "Obesity surgery" OR "Metabolic surgery" OR "Metabolic surgeries" OR "Bariatric surgical procedures" OR "Bariatric surgical procedure" OR "Gastric bypass" OR "Roux-en-Y gastric bypass" OR Gastroplasty OR "Roux en Y Gastric Bypass" OR Sleeve OR "Sleeve gastrectomy" OR "Vertical sleeve")) AND ALL=("Physical activity" OR "Physical activities" OR "Physical fitness" OR "Physical training" OR Exercise OR "Physical exercise" OR "Physical exercises" OR "Exercise training" OR "Exercise trainings" OR "Exercise test" OR "Exercise tests" OR "Physical fitness testing" OR "Fitness testing" OR "Aerobic exercise" OR "Aerobic exercises" OR "Aerobic training program" OR "Exercise therapy" OR "Exercise movement technics" OR "Endurance training" OR "Physical endurance" OR "Performance task" OR "Performance tasks" OR "Performances task" OR "Task performance analysis" OR "Task Performances Analysis" OR "Resistance training" OR "Resistance training intervention" OR "Resistance training program" OR "Strength training" OR "Weight-lifting strengthening program" OR "Weight-lifting strengthening programs" OR "Weight-lifting exercise program" OR "Weight-lifting exercise programs" OR "Weight-bearing strengthening program" OR "Weight-bearing strengthening programs" OR "Weight-bearing exercise program" OR "Weight-bearing exercise programs")) AND ALL=(Strength OR Torque OR "peak torque" OR "Muscle strength" OR "Force production" OR "Physical function" OR "Hand strengths" OR "Hand strength" OR "Muscle contraction" OR "Muscle contractions" OR "Muscular contraction" OR "Muscular contractions" OR "Lean body mass" OR "Muscle mass" OR "Fat free mass" OR "Muscle cross-sectional area" OR "Muscle cross sectional area" OR "Body composition" OR "Body compositions" OR "Body constitution" OR "Body constitutions" OR Anthropometry OR "Body weights Measures" OR "Body measures" OR "Body size" OR "Muscle fatigue" OR "Muscle protein" OR "Muscle proteins" OR "Skeletal muscle" OR "Skeletal muscles" OR "Skeletal muscle mass" OR "Muscle thickness" OR Hypertrophy OR hyperplasia) |
| **PROQUEST** | noft(“Bariatric surgery” OR “Bariatric surgeries” OR “Obesity surgery” OR “Metabolic surgery” OR “Metabolic surgeries” OR “Bariatric surgical procedures” OR “Bariatric surgical procedure” OR “Gastric bypass” OR “Roux-en-Y gastric bypass” OR Gastroplasty OR “Roux en Y Gastric Bypass” OR Sleeve OR “Sleeve gastrectomy” OR “Vertical sleeve”) AND noft(“Physical activity” OR “Physical activities” OR “Physical fitness” OR “Physical training” OR Exercise OR “Physical exercise” OR “Physical exercises” OR “Exercise training” OR “Exercise trainings” OR “Exercise test” OR “Exercise tests” OR “Physical fitness testing” OR “Fitness testing” OR “Aerobic exercise” OR “Aerobic exercises” OR “Aerobic training program” OR “Exercise therapy” OR “Exercise movement technics” OR “Endurance training” OR “Physical endurance” OR “Performance task” OR “Performance tasks“ OR “Performances task” OR “Task performance analysis” OR “Task Performances Analysis” OR “Resistance training” OR “Resistance training intervention” OR “Resistance training program” OR “Strength training” OR “Weight-lifting strengthening program” OR “Weight-lifting strengthening programs” OR “Weight-lifting exercise program” OR “Weight-lifting exercise programs” OR “Weight-bearing strengthening program” OR “Weight-bearing strengthening programs” OR “Weight-bearing exercise program” OR “Weight-bearing exercise programs”) AND noft(Strength OR Torque OR “peak torque” OR “Muscle strength” OR “Force production” OR “Physical function” OR “Hand strengths” OR “Hand strength” OR “Muscle contraction” OR “Muscle contractions” OR “Muscular contraction” OR “Muscular contractions” OR “Lean body mass” OR “Muscle mass” OR “Fat free mass” OR “Muscle cross-sectional area” OR “Muscle cross sectional area” OR “Body composition” OR “Body compositions” OR “Body constitution” OR “Body constitutions” OR Anthropometry OR “Body weights Measures” OR “Body measures” OR “Body size” OR “Muscle fatigue” OR “Muscle protein” OR “Muscle proteins” OR “Skeletal muscle” OR “Skeletal muscles” OR “Skeletal muscle mass” OR “Muscle thickness” OR Hypertrophy OR hyperplasia) |
| **GOOGLE SCHOLAR** | (“Bariatric surgery” OR “Gastric bypass” OR Sleeve) AND (“Physical activity” OR Exercise OR “Aerobic exercise” OR “Resistance training”) AND (Strength OR “Muscle strength” OR “Lean body mass” OR “Muscle mass”) |
