# Supplementary material for: Effect of physical exercise on muscle strength in adults following bariatric surgery: A systematic review and meta-analysis of different muscle strength assessment tests
Source: PLoS One. 2022 Jun 10;17(6):e0269699. doi: 10.1371/journal.pone.0269699 (PMC9187088; doi:10.1371/journal.pone.0269699)
Supplement: S2 Table — (DOCX) [file pone.0269699.s004.docx]

| Studies | Criteria | | | | | | | | | | | | |
| --- | --- | --- | --- | --- | --- | --- | --- | --- | --- | --- | --- | --- | --- |
|  | 1 | 2 | 3 | 4 | 5 | 6 | 7 | 8 | 9 | 10 | 11 | 12 | 13 |
| Stegen et al., 2011 | No | No | Yes | NA | No | No | Yes | Yes | No | Yes | Yes | Yes | Yes |
| Huck, 2015 | No | No | Yes | NA | Yes | No | Yes | Yes | No | No | Yes | Yes | Yes |
| Campanha-Versiani et al., 2017 | No | No | Yes | NA | No | No | Yes | Yes | No | Yes | Unclear | Yes | Yes |
| Coleman et al., 2017 | Yes | Yes | Yes | NA | No | No | Yes | Yes | Yes | Yes | Unclear | Yes | Yes |
| Hassanejad et al., 2017 | Yes | Yes | Yes | NA | No | No | Yes | Yes | No | Yes | Yes | Yes | Yes |
| Herring et al., 2017 | Yes | Yes | Yes | NA | No | No | Yes | Yes | Yes | Yes | Unclear | Yes | Yes |
| Daniels et al., 2018 | Yes | Yes | Yes | NA | No | No | Yes | Yes | Yes | Yes | Yes | Yes | Yes |
| Mundbjerg et al., 2018 | Yes | Yes | Yes | NA | No | No | No | Yes | Yes | Yes | Unclear | Yes | Yes |
| Kelley, 2019 | Yes | Yes | Yes | NA | No | No | No | Yes | Yes | Yes | Yes | Yes | Yes |
| Noack-Segovia et al., 2019 | Yes | Yes | Yes | NA | No | No | Yes | Yes | No | Yes | Yes | Yes | Yes |
| Gallé et al., 2020 | No | No | Yes | NA | Yes | No | Yes | Yes | No | Yes | Unclear | Yes | Yes |
| de Oliveira Junior et al., 2021 | Yes | Unclear | Yes | NA | No | No | Yes | Yes | Yes | Yes | Unclear | Yes | Yes |
| Diniz-Souza et al., 2021 | Yes | Yes | Yes | NA | No | No | No | Yes | Yes | Yes | Yes | Yes | Yes |
| Gil et al., 2021 | Yes | Yes | Yes | NA | No | No | Yes | Yes | Yes | Yes | Yes | Yes | Yes |
| Lamarca et al., 2021 | No | No | Yes | NA | No | No | Yes | Yes | No | Yes | Yes | Yes | Yes |

1 Was true randomization used for assignment of participants to treatment groups?; 2 Was allocation to treatment groups concealed?; 3 Were treatment groups similar at the baseline?; 4 Were participants blind to treatment assignment?; 5 Were those delivering treatment blind to treatment assignment?; 6 Were outcomes assessors blind to treatment assignment?; 7 Were treatment groups treated identically other than the intervention of interest?; 8 Was follow up complete and if not, were differences between groups in terms of their follow up adequately described and analyzed?; 9 Were participants analyzed in the groups to which they were randomized?; 10 Were outcomes measured in the same way for treatment groups?; 11 Were outcomes measured in a reliable way?; 12 Was appropriate statistical analysis used? 13 Was the trial design appropriate, and any deviations from the standard randomized clinical trial design (individual randomization, parallel groups) accounted for in the conduct and analysis of the trial?
