# Supplementary material for: Effect of physical exercise on muscle strength in adults following bariatric surgery: A systematic review and meta-analysis of different muscle strength assessment tests
Source: PLoS One. 2022 Jun 10;17(6):e0269699. doi: 10.1371/journal.pone.0269699 (PMC9187088; doi:10.1371/journal.pone.0269699)
Supplement: S3 Table — (DOCX) [file pone.0269699.s005.docx]

| **Certainty assessment** | | | | | | | **№ of patients** | | **Effect** | | **Certainty** | **Importance** |
| --- | --- | --- | --- | --- | --- | --- | --- | --- | --- | --- | --- | --- |
| **№ of studies** | **Study design** | **Risk of bias^1^** | **Inconsistency^2^** | **Indirectness^3^** | **Imprecision^4^** | **Other considerations** | **Exercise** | **Usual care** | **Relative (95% CI)** | **Absolute (95% CI)** |  |  |
| Muscle Strength - Repetition Maximum test (Upper Limbs) (follow-up: range 12 weeks to 36 weeks) | | | | | | | | | | | | |
| 5 | Randomized and non-randomized trials | very serious^a^ | not serious | serious^b^ | serious^c^ | none | 88 | 83 | - | mean **0.71 higher** (0.41 to 1.01) | ⨁◯◯◯ Very low | CRITICAL |
| Muscle Strength - Repetition Maximum test (Lower Limbs) (follow-up: range 12 weeks to 36 weeks) | | | | | | | | | | | | |
| 6 | Randomized and non-randomized trials | very serious^a^ | serious^d^ | serious^b^ | serious^c^ | none | 70 | 71 | - | mean **1.37 higher** (0.84 to 1.91) | ⨁◯◯◯ Very low | CRITICAL |
| Muscle strength - Sit-to-stand test (follow-up: range 12 weeks to 26 weeks) | | | | | | | | | | | | |
| 10 | Randomized and non-randomized trials | very serious^a^ | very serious^e^ | very serious^b,f^ | serious^c^ | none | 172 | 176 | - | mean **0.60 higher** (0.20 to 1.01) | ⨁◯◯◯ Very low | IMPORTANT |
| Muscle strength - Dynamometer (follow-up: range 12 weeks to 48 weeks) | | | | | | | | | | | | |
| 5 | Randomized and non-randomized trials | very serious^a^ | not serious | very serious^b,f^ | serious^c^ | none | 89 | 72 | - | mean **0.46 higher** (0.06 to 0.87) | ⨁◯◯◯ Very low | CRITICAL |
| Muscle strength - Handgrip (follow-up: range 12 weeks to 54 weeks) | | | | | | | | | | | | |
| 6 | Randomized and non-randomized trials | very serious^a^ | very serious^g^ | serious^b^ | very serious^c,h^ | none | 109 | 128 | - | mean **0.11 higher** (-0.42 to 0.63) | ⨁◯◯◯ Very low | IMPORTANT |

**CI:** confidence interval

#### Explanations

a.High risk of bias studies contribute more than 50% of the weight to the pooled estimate

b.Downgraded one level due high variability of exercise protocols suggested by the included studies

c.Downgraded one level due to total sample size of studies included was lower than 400 participants

d.Downgraded one level due to a moderate (46.14%) and non significant (p=0.09) heterogeneity on Repetition Maximum test (Lower Limb) meta-analysis

e.Downgraded two levels due to a high (68.89%) and significant (p<0.001) heterogeneity on sit-to-stand meta-analysis

f.Downgraded one level due to the heterogeneity of the population related to the post-operative time

g.Downgraded two levels due to a high (73.27%) and significant (p<0.001) heterogeneity, besides a large variability in magnitude of effect on handgrip meta-analysis

h.Downgraded more one level because despite the estimate of treatment effect favors the intervention, 95% confidence interval crossed the central line of null effect in handgrip meta-analysis

#### References

1. Bonner, A., Alexander, P.E., Brignardello-Petersen, R., et al. Applying GRADE to a network meta-analysis of antidepressants led to more conservative conclusions. J. Clin. Epidemiol. ; 2018.

2. Guyatt, G.H., Oxman, A.D., Kunz, R., et al. GRADE guidelines: 7. Rating the quality of evidence—inconsistency. J. Clin. Epidemiol.; 2011.

3. Guyatt, G.H., Oxman, A.D., Kunz, R., et al. GRADE guidelines: 8. Rating the quality of evidence—indirectness. J. Clin. Epidemiol. ; 2011.

4. Guyatt, G.H., Oxman, A.D., Kunz, R., et al. GRADE guidelines: 6. Rating the quality of evidence—imprecision. J. Clin. Epidemiol. ; 2011.
